# Ancestry Prediction Comparisons of Different AISNPs for Five Continental Populations and Population Structure Dissection of the Xinjiang Hui Group via a Self-Developed Panel

**DOI:** 10.3390/genes11050505

**Published:** 2020-05-04

**Authors:** Xiao-Ye Jin, Yu-Xin Guo, Chong Chen, Wei Cui, Yan-Fang Liu, Yun-Chun Tai, Bo-Feng Zhu

**Affiliations:** 1 Key Laboratory of Shaanxi Province for Craniofacial Precision Medicine Research, College of Stomatology, Xi’an Jiaotong University, Xi’an 710004, China; jinxy0901@stu.xjtu.edu.cn (X.-Y.J.); guoyuxin004@163.com (Y.-X.G.); 18883368974@163.com (C.C.); cuiwei3702@163.com (W.C.); 2 College of Forensic Medicine, Xi’an Jiaotong University Health Science Center, Xi’an 710061, China; 3 Clinical Research Center of Shaanxi Province for Dental and Maxillofacial Diseases, College of Stomatology, Xi’an Jiaotong University, Xi’an 710004, China; 4 Multi-Omics Innovative Research Center of Forensic Identification; Department of Forensic Genetics, School of Forensic Medicine, Southern Medical University, Guangzhou 510515, China; liuyanfang92@i.smu.edu.cn (Y.-F.L.); yunchun_tai@163.com (Y.-C.T.)

**Keywords:** ancestry informative markers, SNP, Hui, bio-geographical origins, forensics

## Abstract

Ancestry informative markers are genetic markers that show distinct genetic divergences among different populations. These markers can be utilized to discern population substructures and estimate the ancestral origins of unknown individuals. Previously, we developed a multiplex system of 30 ancestry informative single nucleotide polymorphism (AISNP) loci to facilitate ancestral inferences in different continental populations. In the current study, we first compared the ancestry resolutions of the 30 AISNPs and the other previously reported AISNP panels for African, European, East Asian, South Asian and American populations. Next, the genetic components of the Xinjiang Hui group were further explored in comparison to these continental populations based on the 30 AISNPs. Genetic divergence analyses of the 30 AISNPs in these five continental populations revealed that most of the AISNPs showed high genetic differentiations between these populations. Ancestry analysis comparisons of the 30 AISNPs and other published AISNPs revealed that these 30 AISNPs had comparable efficiency to other AISNP panels. Genetic relationship analyses among the studied Hui group and other continental populations demonstrated that the Hui group had close genetic affinities with East Asian populations and might share the genetic ancestries with East Asian populations. Overall, the 30 AISNPs can be used to predict the bio-geographical origins of different continental populations. Moreover, the obtained genetic data of 30 AISNPs in the Hui group can further enrich the extant reference data, which can be used as reference data for ancestry analyses of the Hui group.

## 1. Introduction

A bio-geographical origin analysis can determine the population substructures in a genome-wide association study [1]. This type of analysis also has wide applications in forensic research. For example, ancestral inferences of unknown individuals may provide valuable information that can assist forensic investigations by narrowing the detection scope; it can also help corroborate eyewitness accounts [2]. In human genome diversity research, forensic geneticists have selected and reported some AISNP and ancestry informative insertion/deletion (InDel) loci for the bio-geographical origin analyses of different continental populations [3,4,5,6]. Phillips et al. developed a multiplex SNP panel for the ancestral analyses of three continental populations (African, European and East Asian) based on the SNaPshot method [4]. They replaced the SNP locus (rs727811) with a highly informative SNP locus (rs3827760) in the following study [7]. Kidd et al. selected and constructed a multiplex system for predicting individual ancestries in 73 populations from eight bio-geographical regions based on TaqMan SNP Genotyping Assays [3]. Wei et al. developed a single-tube 27-plex SNP system for differentiating three continental populations based on the SNaPshot method and then assessed the admixed proportions of four Eurasian populations (Uyghur, Tajik, Kirgiz and Kazak groups) in Xinjiang province based on a self-developed panel [5]. Lan et al. constructed a multiplex InDels panel that could achieve ancestry resolutions of three continental populations [6].

Next generation sequencing (NGS), which refers to massively parallel sequencing, can simultaneously detect a number of genetic markers in an experiment. More importantly, it can provide more information than capillary electrophoresis, such as sequence variations and other genetic variations in the neighboring regions of the targeted markers [8,9]. Recently, some commercial kits based on NGS have been developed, such as the HID-Ion AmpliSeq Identity Panel (Thermo Fisher Scientific, Waltham, MA, USA) for individual identification, the HID-Ion AmpliSeq Ancestry Panel (Thermo Fisher Scientific, Waltham, MA, USA) for forensic ancestry analysis and the ForenSeq DNA Signature Prep Kit (Illumina, San Diego, CA, USA) for ancestry, identity and phenotype research. Altogether, NGS technology shows great potential in forensic science.

The Hui group—the second largest minority group in China—includes more than ten million individuals, according to the report of the sixth nationwide population census. Hui individuals are distributed in many regions, including Ningxia, Xinjiang, Gansu, Yunnan provinces and etc [10]. Previous research indicated that Hui individuals might be ethnic descendants of merchants from Arabia and Persia during the Tang dynasty [11]. Moreover, they might be Muslims from Central Asia and West Asia who were forced to immigrate into China during the Yuan dynasty, coexisted with other ethnic groups and gradually formed the Hui group [12]. Xie et al. assessed the genetic substructures of the Chinese Hui groups residing in different regions based on Y-SNP and Y short tandem repeat (STR) loci. And they found that these Hui groups presented genetic differentiations and could be classified into Hui group in northwest China, Hui groups in Sichuan and Shandong provinces and the Hui group in Yunnan province. Therefore, the authors suggested that different genetic databases should be employed for forensic investigations into these Hui groups [13]. In previous studies, our research team conducted a series of population genetic analyses of Hui group based on autosomal InDels [14], autosomal STR [10,15], Y-STR [16] and X-STR [17] loci and found that the Hui group had close genetic relationships with the Chinese Han populations. However, these genetic markers were mainly used for forensic individual identifications, which might induce some bias in the genetic background analyses of the Hui group. Although the forensic ancestral analysis of the Hui group in Ningxia province was conducted using 165 AISNP loci [18], the genetic architecture of the Hui group in Xinjiang province has not yet been fully explored using ancestral informative markers.

Previously, we selected 30 novel AISNPs for distinguishing African, European, East Asian and South Asian populations and constructed a multiplex system based on the NGS platform [19]. Here, to evaluate the power of the panel to differentiate American populations, we firstly assessed the genetic distributions of the 30 AISNPs in African, European, East Asian, South Asian and American populations. Secondly, we compared the ancestry resolutions of the 30 AISNPs with the other published AISNPs [3,5,7] for these continental populations using a cross-validation procedure. Thirdly, to further enrich the genetic data of the 30 AISNPs in Chinese populations, the genetic information and forensic values of the 30 AISNPs in the Hui group were evaluated. Finally, the genetic structures and phylogenetic relationships between the studied Hui group and these continental populations were further explored based on these 30 AISNPs.

## 2. Materials and Methods

### 2.1. Sample Information

The bloodstain samples of 98 Hui individuals in China were collected with their written informed consent. Twenty-six populations from five continents were used as reference populations; the genetic data for 30 AISNPs in these populations were obtained from the 1000 Genomes Project [20]. This research was carried out in accordance with the Declaration of Helsinki. Moreover, the study protocol was agreed upon by the ethics committee of Xi’an Jiaotong University Health Science Center (2019–1039).

### 2.2. DNA Extraction and Primer Design

DNA samples were isolated from bloodstain cards using a Magbead Blood Spots DNA Kit (CWBio, Beijing, China) according to the manufacturer’s description. The DNA concentration of each sample was determined using a NanoDrop 2000 instrument (Thermo Fisher Scientific, Waltham, MA, USA). The primer designs for the 30 AISNP loci were conducted using the Primer Premier v6.23 software (Premier Biosoft International, Palo Alto, Santa Clara, CA, USA), and then these primers were mixed into the Primer Mix. Primer information of 30 AISNPs was presented in Supplementary Table S1.

### 2.3. Library Preparation, NGS and Data Analysis

The DNA library was prepared by two-rounds PCR. Detailed information of two-rounds PCR was shown in Supplementary Figure S1. Barcode sequences used in this study were given in Supplementary Table S2. And then we determined the library quantification using a Qubit dsDNA HS Assay Kit (Thermo Fisher Scientific, Waltham, MA, USA).

Paired-end sequencing with a read length of 150 bp was conducted on the Illumina NextSeq 500 platform that could produce 100–200 G data in a run. A total of 150 cycles were used to conduct sequencing; other parameters were set according to the manufacturer’s recommendation. Raw data for each individual were saved in the FASTQ format after sequencing. By filtering the sequences with low-quality reads (<80 bp) and sequencing adapters, clean data were obtained from the raw data using the Cutadapt software [21]. After quality control, these data were mapped to the UCSC hg19 human reference genome by the mean algorithm of the BWA software [22] based on the default parameters. Duplicate reads were removed using Picard tools (http://broadinstitute.github.io/picard/), and mapping reads were used to detect variations. Genetic variations of the 30 AISNP loci were detected by the GATK [23] and the mpileup2cns algorithm (min coverage > 30, min var freq > 0.005, *p* value > 0.05, output vcf = 1, min reads > 2) in the VarScan software [24].

### 2.4. Statistical Analysis

The allele coverage ratio (ACR) of each AISNP locus was calculated by the following formula: minor allele coverage/major allele coverage. The Hardy–Weinberg equilibrium (HWE) tests of 30 AISNPs in the Xinjiang Hui group were performed using the Genepop software v4.0 [25] with the probability test described by Guo et al. [26]. The allelic frequency differences (δ), fixation index (*Fst)* and *the* informativeness-for-assignment *(In)* values of the 30 AISNPs between one continent and the other four continents were calculated by the AncestrySNPminer online tool (https://research.cchmc.org/mershalab/AncestrySNPminer). Then, the *Fst* and *In* values of the 30 AISNPs in all continental populations were calculated according to the previous report [2]. By selecting “Hardy–Weinberg principle applies to your marker set”, verbose cross-validation analyses of the five continental populations were conducted through the “Thorough analysis of population data” option in the Snipper v2.5 (http://mathgene.usc.es/snipper/). The correlation coefficient *r*^2^ values of pairwise AISNP loci in the Hui group were estimated by the Haploview software v4.2 [27]. The heterozygosity values and minor allelic frequencies (MAF) of 30 AISNPs in the Hui group were also calculated by the Haploview software v4.2. The forensic parameters of 30 AISNPs in the Hui group were estimated by the PowerStats program (Promega, Madison, WI, USA).

The population genetic relationships between the Hui group and the 26 reference populations from five continents were determined based on the 30 AISNPs. Principal component analyses (PCAs) of the Hui group and different continental populations were performed using the XLSTAT program (https://www.xlstat.com). Nei’s *D_A_* distances among the Hui group and 26 reference populations were estimated by the DISPAN software [28]. A neighbor-joining tree of the Hui group and reference populations was reconstructed by the MEGA software v6.0 [29], based on their Nei’s *D_A_* values. The pairwise *Fst* values of the Hui group and other reference populations were calculated using the Genepop software v4.0, and the heatmap of pairwise *Fst* values was built with the *R* software v3.3 [30]. Based on the ADMIXTURE software v1.3 [31], the genetic structure analyses among the Hui group and other reference populations were performed for each of the *K* values 2–6; the detailed parameters used in the ADMIXTURE software were as follows: we used the block relaxation algorithm (optimization method), and the log-likelihood increased by less than 10^−^^4^ (termination criteria). Then, the estimated ancestral components of these populations were displayed as a bar plot by the CLUMPAK online tool [32].

## 3. Results and Discussion

### 3.1. Depth of Coverage and the ACR of the 30 AISNPs in the Hui Group

**Figure 1:**
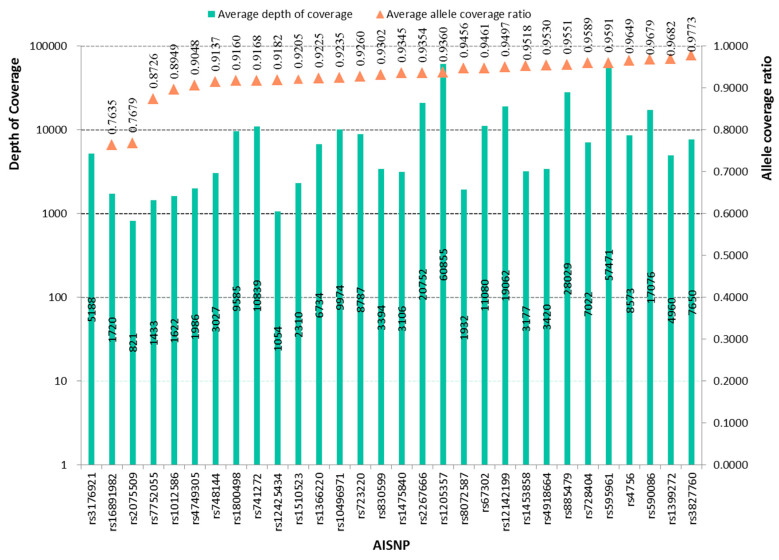
Depth of coverage and allele coverage ratios of 30 AISNPs in the Hui group.

Depth of coverage (DOC) that is the number of sequencing target regions is usually used as the metric to evaluate the data generated from massively parallel sequencing. For the ForenSeq^TM^ DNA Signature Prep Kit, more than 20 reads were considered as the detection threshold for the sequencing data of 230 genetic markers in previous research [33]. Guo et al. additionally proposed that more than 30 reads could be used as the interpretation threshold to aid in the analyses of loci with heterozygote alleles [34]. In this study, we detected the genetic profiles of 30 AISNPs in the Hui group using the NGS. The average DOC values for the 30 AISNPs in the Hui group ranged from 821 (rs2075509) to 60,855 (rs1205357), as shown in Figure 1. The lowest DOC value was observed at the rs723220 locus with a DOC value of 30 (data not shown), which was more than the detection threshold and equal to the interpretation threshold mentioned above.

ACR can evaluate the heterozygosity balance or intralocus balance: the locus that has a higher ACR is more beneficial for mixture analysis [35]. Since the rs3176921 locus showed homozygous alleles in all Hui individuals, the ACR of this locus was not estimated in the following study. The average ACR values of the remaining 29 AISNPs were also shown in Figure 1. The results revealed a range from 0.7635 to 0.9773, indicating that these 29 AISNPs had a good intralocus balance. Therefore, most of the 30 AISNPs may be useful to disentangle the mixtures based on the obtained ACR values, which remained to be further validated in future research.

### 3.2. Genetic Divergences of the 30 AISNPs among the Five Continental Populations

**Figure 2:**
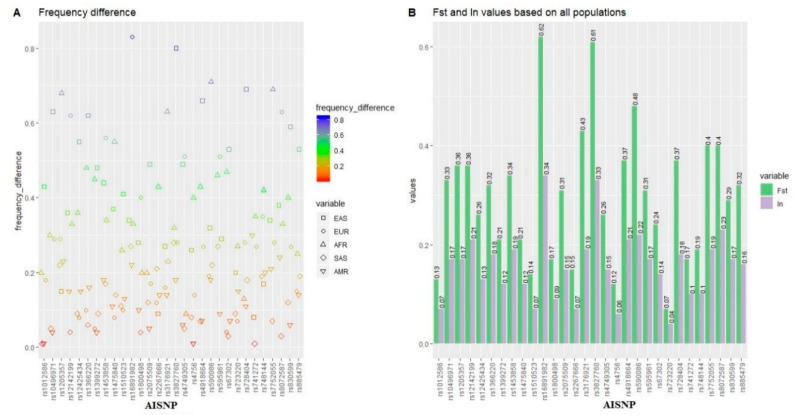
Genetic divergences of 30 AISNPs in five continental populations. (**A**). Frequency differences of 30 AISNPs between one continent and the other four continents. (**B**). Overall, *Fst* and *In* values of 30 AISNPs in all continental populations. AFR, African; AMR, American; EAS, East Asian; EUR, European; SAS, South Asian.

Based on the genetic data of the five continental populations in the 1000 Genomes Project, we evaluated the genetic divergences of these AISNPs among these continental populations. δ values are the allelic frequency differences of the genetic markers in different populations, which can measure the genetic divergences of the markers [2]. Generally speaking, a locus with a high δ value is suitable as the AIM for ancestry analyses. First, the δ values of 30 AISNPs in one continent vs. the other four continents were estimated, as shown in Figure 2A. We found that 15 out of 30 AISNPs showed large δ values (>0.40) between East Asian populations and the other continental populations. There were, moreover, six and 12 AISNPs with large δ values (>0.40) in European populations vs. the remaining populations, and African populations vs. the remaining populations, respectively. Relatively large δ values (>0.20) in South Asian vs. other continental populations, and American vs. other continental populations were observed at seven, and five loci, respectively. Besides δ values, the *Fst* and *In* values can also be used as the metrics for evaluating the power of genetic markers to distinguish different populations [2]. Thus, we also calculated the pairwise *Fst* and *In* values of 30 AISNPs in these continental populations, as shown in Supplementary Figure S2. The loci that showed high δ values in pairwise populations had high *Fst* and *In* values. Furthermore, relatively low *Fst* and *In* values (<0.10) in South Asian vs. other continental populations, and American vs. other continental populations were observed for most AISNP loci, indicating that these AISNPs might have lower divergences between South Asian/American populations and other continental populations. The *Fst* and *In* values of the 30 AISNPs among all continental populations were shown in Figure 2B. The *Fst* and *In* values ranged from 0.07 to 0.62, and from 0.04 to 0.34, respectively.

### 3.3. Ancestry Resolution Comparisons of Different AISNPs among Five Continental Populations

The cross-validation analysis in the Snipper software can re-estimate the allelic frequencies of genetic markers in training populations after randomly removing one sample successively and then can infer the ancestral origin of the removed individual based on the remaining dataset. This analysis can evaluate the values of a set of novel AISNPs to infer ancestry [36]. Cross-validation analyses of five continental populations were conducted based on the 30 studied AISNPs and previously published AISNPs [3,5,7], as presented in Table 1. For the 30 studied AISNPs, most individuals from the five continents could be assigned to their corresponding continental origins. However, we found that some individuals were classified into other continental populations, particularly for American populations. Among these four AISNP panels (Table 1), the 55 AISNPs selected by Kidd et al. provided the best ancestry resolution performance, even for admixed American populations, followed by the 33 AISNPs. Nonetheless, we found that the 30 studied AISNPs displayed slightly higher accuracy for the ancestry analyses of African and East Asian populations than the 33 AISNPs. Given the results in Table 1, the 30 studied AISNPs were able to achieve ancestry analyses of the four continental populations (African, East Asian, European and South Asian), which further corroborated the previous findings [19]. We also noted that these 30 AISNPs performed less efficiently in differentiating American populations from other continental populations. The American populations collected by the 1000 Genomes Project possessed different proportions of ancestries from European, African and indigenous American populations, which might create challenges for the ancestry analyses of American populations. Therefore, the power of the 30 studied AISNPs to differentiate American populations from other continental populations should be further evaluated with indigenous American individuals. Moreover, those highly informative SNPs, which are useful for the ancestral analyses of American populations, should be incorporated into the developed AISNP panel in the future.

### 3.4. Genetic Distributions and Forensic Parameters of 30 AISNPs in the Hui Group

The *p*-values of HWE tests for 30 AISNPs in the Hui group were presented in Supplementary Table S3. Since the rs3176921 locus showed homozygous alleles in all studied Hui individuals, the HWE test for the locus was not conducted. For the other 29 AISNP loci, we found that the *p*-values of these AISNPs were larger than 0.05, except for the rs590086 locus. Nonetheless, these 29 AISNP loci conformed to HWE in the Hui group after applying Bonferroni correction (*p* = 0.05/29 = 0.0017). We also described the results of linkage disequilibrium analyses of 30 AISNPs in the studied Hui group, revealing that the pairwise *r*^2^ values of these AISNPs were less than 0.1 (Supplementary Figure S3). These relatively low *r*^2^ values indicated that these AISNPs had weak correlations and could be viewed as independent loci from each other in the Hui group.

**Figure 3:**
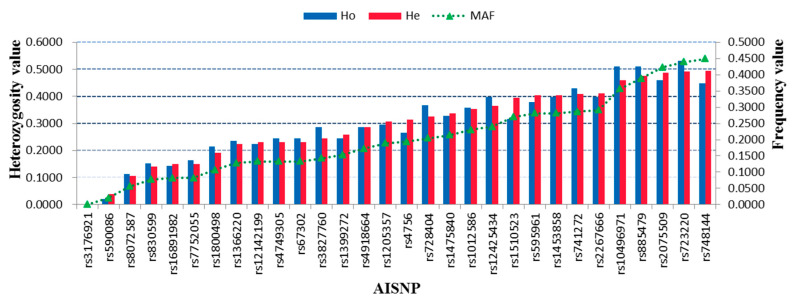
Heterozygosity values and MAF of 30 AISNPs in the Hui group. Ho, observed heterozygosity; He, expected heterozygosity.

The SNP locus can be viewed as a valuable marker for forensic individual identification once its MAF is greater than 0.2 [37]. As shown in Figure 3, the findings for the MAF of the 30 AISNP loci revealed that there were 14 AISNP loci with MAF greater than 0.2. Furthermore, we also evaluated the heterozygosity of 30 AISNPs in the Hui group (Figure 3 and Supplementary Table S3). The results showed that observed heterozygosity (Ho) and expected heterozygosity (He) of the 30 AISNPs in the Hui group ranged from 0.0000 (rs3176921) to 0.5310 (rs723220), and from 0.0000 (rs3176921) to 0.4950 (rs748144), respectively. Nine out of 30 AISNPs showed relatively high He values (>0.4), suggesting that these loci could be used as individual identification SNPs for forensic applications in the Hui group. The forensically relevant parameters of the 30 AISNPs in the studied group were given in Supplementary Table S3. The average matching probability, power of discrimination (PD), polymorphism information content and power of exclusion (PE) values of the 30 AISNPs in the studied Hui group were 0.5653, 0.4347, 0.2441 and 0.0782, respectively. The cumulative power of discrimination (CPD) and power of exclusion (CPE) values in the studied Hui group were 0.999 999 987 and 0.9183, respectively. Compared to the results of the 30 InDels [14] and STRs [10,15] in the Hui group, these 30 AISNPs were of less value for individual identification and paternity testing. Nevertheless, the CPD value (0.999 999 987) of the 30 AISNPs in the Hui group demonstrated that these AISNPs could also be used as a supplementary tool for forensic individual identification.

### 3.5. Phylogenetic Relationships and Population Structure Analyses of the Hui Group and Other Continental Populations

**Figure 4:**
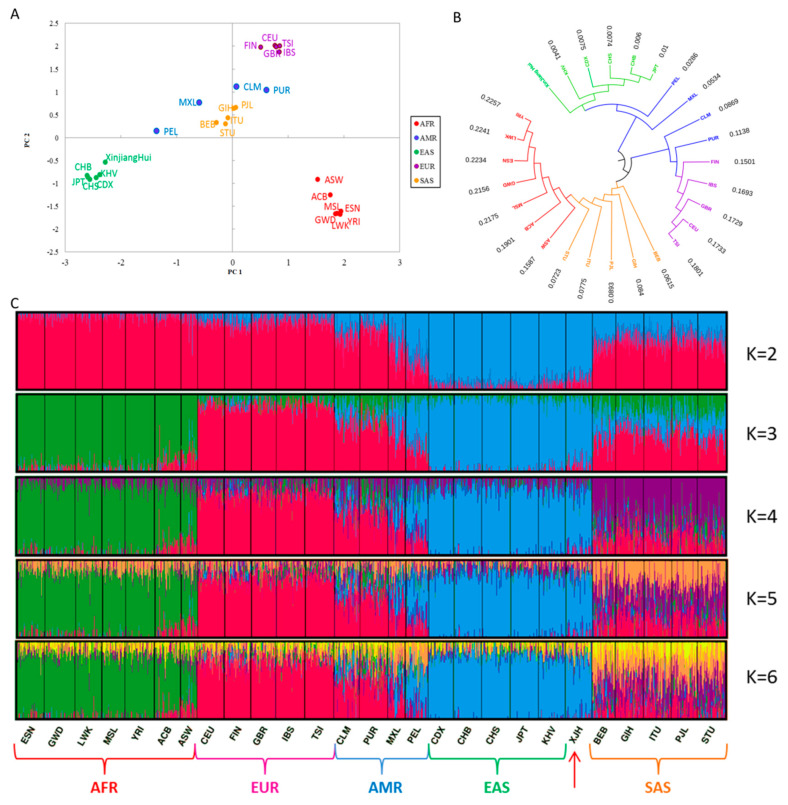
Population genetic relationship analyses of Xinjiang Hui group and other continental populations. (**A**). principal component analysis of these populations at PC1 and PC2. (**B**). phylogenetic tree of these populations; numeric indicated Nei’s *D_A_* values between Hui group and other continental populations. (**C**). population genetic structure analyses of these populations at *K* = 2–6. AFR, African; AMR, American; EAS, East Asian; EUR, European; SAS, South Asian; ESN, Esan in Nigeria; GWD, Gambian in Western Division; LWK, Luhya in Webuye, Kenya; MSL, Mende in Sierra Leone; YRI, Yoruba in Ibadan; ACB, African Caribbean in Barbados; ASW, African Ancestry in Southwest US; CEU, Utah residents (CEPH) with Northern and Western European ancestry; FIN, Finnish in Finland; GBR, British in England and Scotland; IBS, Iberian populations in Spain; TSI, Toscani in Italy; CLM, Colombian in Medellin; PUR, Puerto Rican in Puerto Rico; MXL, Mexican Ancestry in Los Angeles; PEL, Peruvian in Lima; CDX, Chinese Dai in Xishuangbanna; CHB, Han Chinese in Beijing; CHS, Han Chinese South; JPT, Japanese in Tokyo; KHV, Kinh in Ho Chi Minh City, Vietnam; XJH, Xinjiang Hui; BEB, Bengali in Bangladesh; GIH, Gujarati Indian in Houston; ITU, Indian Telugu in the UK; PJL, Punjabi in Lahore; STU, Sri Lankan Tamil in the UK.

Based on the 30 selected AISNPs, we explored the population genetic relationships among the studied Hui group and other continental populations using multiple methods. PCA is one of the multivariate analysis methods; it can extract the most important variables that account for most information in the raw dataset. By reducing the dimensionality of the dataset, the studied subjects can be graphically represented in a two-dimensional plane, which can visually recognize the relationships between these subjects [36]. Thus, we first conducted a PCA of the studied Hui group and other continental populations at PC1 and PC2, as shown in Figure 4A. We found that the populations from the four continents (African, European, East Asian and South Asian) formed four clusters, and the populations in the same continent clustered together; the American populations distributed among European, East Asian and South Asian populations. The PCA for these populations was also conducted at an individual level (Supplementary Figure S4), which showed similar distribution patterns. For the studied Hui group, most individuals were overlapped on the East Asian individual cluster. Nei’s *D_A_* distance refers to the genetic distances (related to mutation and genetic drift) between pairwise populations. The *D_A_* distance can give a reliable population phylogenetic tree [38]. Next, we constructed a phylogenetic tree among the Hui group and other populations based on Nei’s *D_A_* distances, as shown in Figure 4B. Three apparent branches could be seen from the phylogenetic tree: five East Asian populations, the studied Hui group, and two American populations (Peruvian in Lima and Mexican Ancestry in Los Angeles) were located in the same branch. The four European populations and the other two American populations (Colombian in Medellin and Puerto Rican in Puerto Rico) were located in one branch, while African and South Asian populations were positioned in another branch. Moreover, our findings for Nei’s *D_A_* distances demonstrated that the Hui group featured minor genetic differences from East Asian populations. We also assessed the pairwise *Fst* values of Hui group and other continental populations, as shown in Supplementary Figure S5. We found that the Hui group had relatively small *Fst* values compared to East Asian populations. The ADMIXTURE software can discern a population’s substructure, estimate ancestry components, and study the admixtures between populations [31]. To further dissect the population structure of the studied Hui group, a genetic structural analysis of the Hui group was conducted in comparison to the other continental populations using the ADMIXTURE software (Figure 4C). Firstly, with an increase in the *K* values, populations from the same continent showed similar genetic component distributions. However, American populations showed admixed ancestral proportions of European and East Asian populations. Secondly, no further distinctions could be made between the Hui group and East Asian populations, revealing close genetic affinities between Hui group and these East Asian populations.

Yao et al. investigated the genetic structure of the Hui group residing in Gansu province via autosomal STR loci and found that the Hui group might have common genetic ancestry with East Asian populations [39]. He et al. comprehensively explored the genetic background and ancestry components of the Hui group from the Ningxia region and found that the Hui group had close genetic relationships with Chinese Han populations that showed prominent East Asian ancestry components [18]. Zhou et al. explored the admixture signals of the Ningxia Hui group based on a set of InDels and found that the East Asian populations provided greater genetic contributions to the Ningxia Hui group than western Eurasian populations [40]. For the Xinjiang Hui group, similar conclusions were made in our previous research [10,14,15]. Here, we further dissected the genetic components of the Xinjiang Hui group based on a set of AISNP loci. The obtained results provided evidence for the East Asian origin of the studied Hui group, which might be related to the greater gene flow between the Hui group and East Asian populations. However, genetic distribution analyses of the genetic markers on the Y chromosomes in the Hui groups from different regions revealed their genetic substructures [13]. Therefore, further analyses of the genetic components in the Hui groups from other regions should be conducted based on the developed AISNP panel.

## 4. Conclusions

In this study, we compared the ancestry resolutions of previously selected 30 AISNPs and other published AISNP panels and found that studied 30 AISNPs could be used for ancestry analyses of African, East Asian, European and South Asian populations. The obtained population data of 30 AISNPs in the Xinjiang Hui group can be employed as reference data for ancestry origin analysis of the Hui group. Furthermore, population genetic analysis between the studied Hui group and other continental populations based on 30 AISNPs revealed that the Hui group might have similar ancestry origins with East Asian populations.

## Figures and Tables

**Table 1:** Ancestry resolution comparisons of the studied 30 AISNPs and other previously published AISNP panels among five continental populations.

Panels	Intercontinental Populations	AFR	AMR	EAS	EUR	SAS
this study	AFR	**0.9864**	0.0076	0.0000	0.0015	0.0045
	AMR	0.0058	**0.6858**	0.0605	0.1816	0.0663
	EAS	0.0000	0.0000	**1.0000**	0.0000	0.0000
	EUR	0.0000	0.0139	0.0000	**0.9801**	0.0060
	SAS	0.0000	0.0736	0.0000	0.0143	**0.9121**
55 AISNPs	AFR	**0.9924**	0.0076	0.0000	0.0000	0.0000
	AMR	0.0058	**0.8069**	0.0029	0.1527	0.0317
	EAS	0.0000	0.0000	**1.0000**	0.0000	0.0000
	EUR	0.0000	0.0000	0.0000	**1.0000**	0.0000
	SAS	0.0000	0.0102	0.0000	0.0041	**0.9857**
33 AISNPs	AFR	**0.9849**	0.0151	0.0000	0.0000	0.0000
	AMR	0.0086	**0.7983**	0.0375	0.1124	0.0432
	EAS	0.0000	0.0040	**0.9940**	0.0000	0.0020
	EUR	0.0000	0.0159	0.0000	**0.9801**	0.0040
	SAS	0.0000	0.0409	0.0000	0.0061	**0.9530**
27 AISNPs	AFR	**0.9879**	0.0091	0.0000	0.0000	0.0030
	AMR	0.0058	**0.6426**	0.0778	0.1499	0.1239
	EAS	0.0000	0.0020	**0.9980**	0.0000	0.0000
	EUR	0.0000	0.0119	0.0000	**0.9801**	0.0080
	SAS	0.0000	0.1493	0.0000	0.0429	**0.8078**

Note: The rs1321333 locus of 34 AISNP panel developed by Fondevila et al. [7] was not reported in the 1000 Genomes Project phase III. Therefore, cross-validation analyses of five continental populations were conducted using 33 AISNPs. Numeric in bold indicated correctly assigned proportions of continental populations. AFR, African; AMR, American; EAS, East Asian; EUR, European; SAS, South Asian.

## Data Availability

Genetic data of 30 AISNPs in Xinjiang Hui group were only used as scientific research. Data in this study are available from the corresponding author. Personal information of participants will not be shared with any individuals or organizations.

## Supplementary Materials

The following are available online at https://www.mdpi.com/2073-4425/11/5/505/s1, Supplementary Figure S1: Detailed information of two rounds PCR used for library preparation. Supplementary Figure S2: *Fst* (A) and *In* (B) values of 30 AISNPs between one continent and the other four continents. AFR, African; AMR, American; EAS, East Asian; EUR, European; SAS, South Asian. Supplementary Figure S3: Linkage disequilibrium analyses (*r*^2^) of pairwise 30 AISNPs in Xinjiang Hui group. To better show *r*^2^ values, these *r*^2^ values were expanded to 100 times. Supplementary Figure S4: Principal component analysis of Xinjiang Hui group and other continental populations at individual level. AFR, African; AMR, American; EAS, East Asian; EUR, European; SAS, South Asian; XJH, Xinjiang Hui. Supplementary Figure S5: The heatmap of *Fst* values among Hui group and other continental populations. AFR, African; AMR, American; EAS, East Asian; EUR, European; SAS, South Asian; ESN, Esan in Nigeria; GWD, Gambian in Western Division; LWK, Luhya in Webuye, Kenya; MSL, Mende in Sierra Leone; YRI, Yoruba in Ibadan; ACB, African Caribbean in Barbados; ASW, African Ancestry in Southwest US; CEU, Utah residents (CEPH) with Northern and Western European ancestry; FIN, Finnish in Finland; GBR, British in England and Scotland; IBS, Iberian populations in Spain; TSI, Toscani in Italy; CLM, Colombian in Medellin; PUR, Puerto Rican in Puerto Rico; MXL, Mexican Ancestry in Los Angeles; PEL, Peruvian in Lima; CDX, Chinese Dai in Xishuangbanna; CHB, Han Chinese in Beijing; CHS, Han Chinese South; JPT, Japanese in Tokyo; KHV, Kinh in Ho Chi Minh City, Vietnam; XJH, Xinjiang Hui; BEB, Bengali in Bangladesh; GIH, Gujarati Indian in Houston; ITU, Indian Telugu in the UK; PJL, Punjabi in Lahore; STU, Sri Lankan Tamil in the UK. Supplementary Table S1: Primer information and amplicon lengths of 30 AISNPs. Supplementary Table S2: Barcode sequences used in this study. Supplementary Table S3: Forensic parameters of 30 AISNP loci in the Xinjiang Hui group.
