# Supplementary material for: Ancestry Prediction Comparisons of Different AISNPs for Five Continental Populations and Population Structure Dissection of the Xinjiang Hui Group via a Self-Developed Panel
Source: Genes (Basel). 2020 May 4;11(5):505. doi: 10.3390/genes11050505 (PMC7288656; doi:10.3390/genes11050505)
Supplement: Supplementary file 1 [file genes-11-00505-s001.zip › genes-734405-supplementary/Supplementary Table S1.docx]

Supplementary Table S1. Primer information and amplicon lengths of 30 AISNPs.

| Loci | Amplicon lengths (bp) | Forward primers (5′→3′) | Reverse primers (5′→3′) |
| --- | --- | --- | --- |
| rs12142199 | 147 | GAAGGCCTTGATGTGCTTGAAC | GAACCTGAAGGTGCCCATCTAC |
| rs885479 | 136 | TGGACCGCTACATCTCCATCTT | GTGGTCGTAGTAGGCGATGAAG |
| rs3827760 | 274 | CTCAATCTGCACCAGTTTTGTGA | CTGTGACTTGCAACATCTAACTGTG |
| rs1475840 | 274 | TGAGCTTCAGACATTGTGGCAA | AGTCCTTTGGGAAAGTCATTGTCTATG |
| rs16891982 | 274 | AATCAGTGAGGAAATGACACCTAGAATTC | GAAAGAGGAGTCGAGGTTGGAT |
| rs728404 | 274 | ACACTCACGCATCCTATGTCTG | ACGTTGAGGTTTATGTGTGTGAGT |
| rs1366220 | 274 | TCCATCCCTGCATTAGTCAGGT | AGCAGGGTCTTCAGGTTTCCTA |
| rs4749305 | 274 | AGAATCATACCACATAGTGTACTTTCACAC | CCCTACAAAGTGGCAAACCAAG |
| rs1453858 | 274 | ACTGATGTTTCCCATTCCTCATCATC | ATTAAAGTTTCAAGATGTTGGTGCCAATT |
| rs590086 | 273 | CCTCTCCTGGAGATGGGTTTCA | GCCAGCGAAGCAAATGCAT |
| rs10496971 | 274 | AGAAACAAAACAGAACTGAAAAGCTTCC | AAGAGAATAAAACATCAAACCTACTCAGCA |
| rs4918664 | 274 | CTACTGCTGGTTCCTGGGAATC | GACCAAAAGATTCCTCTTTGGAGGA |
| rs1800498 | 274 | ACATCCCATGCTCTCTACTAAGCT | AGCATTAAGGAATGATGCCTGGAT |
| rs595961 | 211 | CCCTACCCACCTGACTCTACTG | CCTACAGCGAACCCTCCTTACT |
| rs723220 | 110 | CATGTGGTTGCCAAGAATGA | GCTCACGAAGTGGAGTGATGTC |
| rs1012586 | 274 | TAAACCAGGAAGCACAGCTGTT | AGGAAGGGAGAGCAAATGATAACTTAAAAT |
| rs1510523 | 274 | TCTTGCCAAGTGTGAAATAATGAGTTTATG | GCAAGAGTAGGCACTTACAATACACTATG |
| rs12425434 | 274 | CATCCCATTGTTAGAGATGAGGAAGA | GTGATTTCCTTCCTTCATACTCGTTTCTAA |
| rs748144 | 274 | CCTCTCATGCTTTCTCTCTCTCTTATG | GCAATGGCTTAAAGCACACATTATGG |
| rs741272 | 274 | GAAAGGCAGAGATGAGTGTCCA | GTCAGTTGAAATAGATTACAAGAGGGATCT |
| rs67302 | 274 | AGGCCTACAGATTGTGTCTGACATA | GGCTCTCTGGAATACAGATGGGAT |
| rs8072587 | 274 | GTGTTTTTAAGAATTTCATGACAGGGTGAT | GTTTTCCTCTTTGAGAGAAACTAAAAGGTT |
| rs4756 | 238 | GCTGAACACCTTGCCACATACA | GCCTTTCTGGCCAGATCCAATC |
| rs1205357 | 231 | GCCAAAGCTGTGTCCTTTGTTA | GTATCAGGCAGGTGAGCTGTATG |
| rs830599 | 274 | GTGTACAAGGCAGGGCATGTAT | AGGAAAGACAATTAGACAGTGTTCTTTGT |
| rs1399272 | 274 | ATGAAGTGGCTTAAAAATCATAGACCTCA | CCTATTGTCTACCACATCCAACCA |
| rs2267666 | 274 | GCAGAGTTGCTGGCCTAGAAAA | TTCAGGGAGAAGGGCCTAAACCT |
| rs7752055 | 274 | AGTTGAGAAGACTGAATTCACTGTTGAA | GATATAGGGAAAGAAAGAAAGGGAAGCTTT |
| rs3176921 | 274 | GACAGAGTCCCACCATCTTTCTG | GCAGAGAGTTTCTGAGATAACCTAAAATCT |
| rs2075509 | 274 | CTTACCCTCATCTTCCAACATAAATTCTCA | GAAGGCAGAGAAAGAGGCCTTTTA |
