# Supplementary material for: Ancestry Prediction Comparisons of Different AISNPs for Five Continental Populations and Population Structure Dissection of the Xinjiang Hui Group via a Self-Developed Panel
Source: Genes (Basel). 2020 May 4;11(5):505. doi: 10.3390/genes11050505 (PMC7288656; doi:10.3390/genes11050505)
Supplement: Supplementary file 1 [file genes-11-00505-s001.zip › genes-734405-supplementary/Supplementary Table S2.docx]

Supplementary Table S2. Barcode sequences used in this study.

| Samples | Barcodes | Samples | Barcodes | Samples | Barcodes | Samples | Barcodes |
| --- | --- | --- | --- | --- | --- | --- | --- |
| Hui1 | ACTGATAT | Hui26 | TAGTCTAT | Hui51 | TGCAAGAT | Hui76 | AGTAGCGC |
| Hui2 | TCGCGACA | Hui27 | CTGTACAT | Hui52 | GTTGTTAT | Hui77 | TCGCGACA |
| Hui3 | GCACGTAT | Hui28 | GACTGTAT | Hui53 | TGTTTGAT | Hui78 | TGTTGAAT |
| Hui4 | TGTTGAAT | Hui29 | TAATATAT | Hui54 | GTCATAAT | Hui79 | CAGGATAT |
| Hui5 | CAGGATAT | Hui30 | CATCTGAT | Hui55 | AGACTCAT | Hui80 | CATATGAT |
| Hui6 | CATATGAT | Hui31 | TAACTGAT | Hui56 | ATCTAAAT | Hui81 | ATGGTCAT |
| Hui7 | TGGCGCAT | Hui32 | CTGGCAAT | Hui57 | TAGTTGAT | Hui82 | GTCGTAAT |
| Hui8 | CTCACGAT | Hui33 | TGCTAAAT | Hui58 | GTCAGAAT | Hui83 | CTACCTAT |
| Hui9 | ATGGTCAT | Hui34 | GTTCTGAT | Hui59 | AGGGTAAT | Hui84 | CTGTCGAT |
| Hui10 | CGTGAAAT | Hui35 | AGTCTAAT | Hui60 | TGCCGGAT | Hui85 | TGATTAAT |
| Hui11 | GTCGTAAT | Hui36 | GCAATAAT | Hui61 | CTGCCCAT | Hui86 | TCCGCGAT |
| Hui12 | CTACCTAT | Hui37 | AGTCCTAT | Hui62 | GTGTGGAT | Hui87 | CCGTGTAT |
| Hui13 | CTGTCGAT | Hui38 | GTCAGTAT | Hui63 | ATTTGGAT | Hui88 | CGACCGAT |
| Hui14 | TGATTAAT | Hui39 | GAATAAAT | Hui64 | CCATCAAT | Hui89 | TCTGCAAT |
| Hui15 | TCCGCGAT | Hui40 | GTAACCAT | Hui65 | GTACCAAT | Hui90 | ATACTTAT |
| Hui16 | TATCATAT | Hui41 | GCTAGCAG | Hui66 | GGATATAT | Hui91 | GGACTCAT |
| Hui17 | CCGTGTAT | Hui42 | GTCGTTAT | Hui67 | CGTCATAT | Hui92 | CTTGGTAT |
| Hui18 | GTGTTAAT | Hui43 | GTCTGGAT | Hui68 | GTTGGTAT | Hui93 | CTGTACAT |
| Hui19 | CGACCGAT | Hui44 | ATGTAAAT | Hui69 | AATTCAAT | Hui94 | GACTGTAT |
| Hui20 | TCTGCAAT | Hui45 | ACTGTAAT | Hui70 | TGTGGCAT | Hui95 | TAACTGAT |
| Hui21 | ATACTTAT | Hui46 | GCTATGAT | Hui71 | GTCCGGAT | Hui96 | GTTCTGAT |
| Hui22 | GTCAGGAT | Hui47 | CAGCAAAT | Hui72 | AGGACTAT | Hui97 | TGTGGCAT |
| Hui23 | GGACTCAT | Hui48 | TACACAAT | Hui73 | CCGGGAAT | Hui98 | GTTCGCAT |
| Hui24 | CTTGGTAT | Hui49 | GGCAGGAT | Hui74 | GCATCGAG |  |  |
| Hui25 | CAATCAAT | Hui50 | TAACCCAT | Hui75 | ATCTCAGA |  |  |
