# Supplementary material for: Ancestry Prediction Comparisons of Different AISNPs for Five Continental Populations and Population Structure Dissection of the Xinjiang Hui Group via a Self-Developed Panel
Source: Genes (Basel). 2020 May 4;11(5):505. doi: 10.3390/genes11050505 (PMC7288656; doi:10.3390/genes11050505)
Supplement: Supplementary file 1 [file genes-11-00505-s001.zip › genes-734405-supplementary/Supplementary Table S3.docx]

| Supplementary Table S3. Forensic parameters of 30 AISNP loci in the studied Hui group. | | | | | | |  |  |
| --- | --- | --- | --- | --- | --- | --- | --- | --- |
| Loci | MAF | Ho | He | MP | PD | PIC | PE | *P_HWE_* |
| rs1012586 | 0.2300 | 0.3570 | 0.3540 | 0.4804 | 0.5196 | 0.2912 | 0.0899 | 1.0000 |
| rs10496971 | 0.3570 | 0.5100 | 0.4590 | 0.4211 | 0.5789 | 0.3538 | 0.1966 | 0.3784 |
| rs1205357 | 0.1890 | 0.2960 | 0.3060 | 0.5292 | 0.4708 | 0.2594 | 0.0619 | 0.7418 |
| rs12142199 | 0.1330 | 0.2240 | 0.2300 | 0.6210 | 0.3790 | 0.2036 | 0.0368 | 0.6715 |
| rs12425434 | 0.2400 | 0.3980 | 0.3650 | 0.4750 | 0.5250 | 0.2981 | 0.1127 | 0.5778 |
| rs1366220 | 0.1280 | 0.2350 | 0.2230 | 0.6254 | 0.3746 | 0.1978 | 0.0399 | 1.0000 |
| rs1399272 | 0.1530 | 0.2450 | 0.2590 | 0.5858 | 0.4142 | 0.2257 | 0.0432 | 0.6922 |
| rs1453858 | 0.2810 | 0.3980 | 0.4040 | 0.4359 | 0.5641 | 0.3222 | 0.1127 | 1.0000 |
| rs1475840 | 0.2140 | 0.3270 | 0.3370 | 0.4967 | 0.5033 | 0.2800 | 0.0750 | 0.7653 |
| rs1510523 | 0.2700 | 0.3160 | 0.3950 | 0.4392 | 0.5608 | 0.3167 | 0.0705 | 0.0696 |
| rs16891982 | 0.0820 | 0.1430 | 0.1500 | 0.7378 | 0.2622 | 0.1387 | 0.0161 | 0.4861 |
| rs1800498 | 0.1070 | 0.2140 | 0.1910 | 0.6633 | 0.3367 | 0.1730 | 0.0338 | 0.5943 |
| rs2075509 | 0.4230 | 0.4590 | 0.4880 | 0.3688 | 0.6312 | 0.3691 | 0.1542 | 0.5407 |
| rs2267666 | 0.2910 | 0.3980 | 0.4120 | 0.4271 | 0.5729 | 0.3274 | 0.1127 | 0.8062 |
| rs3176921 | 0.0000 | 0.0000 | 0.0000 | 1.0000 | 0.0000 | 0.0000 | 0.0000 | - |
| rs3827760 | 0.1430 | 0.2860 | 0.2450 | 0.5918 | 0.4082 | 0.2149 | 0.0578 | 0.2060 |
| rs4749305 | 0.1330 | 0.2450 | 0.2300 | 0.6150 | 0.3850 | 0.2036 | 0.0432 | 1.0000 |
| rs4756 | 0.1940 | 0.2650 | 0.3130 | 0.5277 | 0.4723 | 0.2637 | 0.0502 | 0.1878 |
| rs4918664 | 0.1730 | 0.2860 | 0.2870 | 0.5500 | 0.4500 | 0.2456 | 0.0578 | 1.0000 |
| rs590086 | 0.0200 | 0.0200 | 0.0400 | 0.9402 | 0.0598 | 0.0392 | 0.0004 | 0.0307 |
| rs595961 | 0.2810 | 0.3780 | 0.4040 | 0.4325 | 0.5675 | 0.3222 | 0.1008 | 0.6155 |
| rs67302 | 0.1330 | 0.2450 | 0.2300 | 0.6150 | 0.3850 | 0.2036 | 0.0432 | 1.0000 |
| rs723220 | 0.4390 | 0.5310 | 0.4930 | 0.3992 | 0.6008 | 0.3712 | 0.2157 | 0.5399 |
| rs728404 | 0.2040 | 0.3670 | 0.3250 | 0.5102 | 0.4898 | 0.2721 | 0.0953 | 0.3469 |
| rs741272 | 0.2860 | 0.4290 | 0.4080 | 0.4388 | 0.5612 | 0.3249 | 0.1323 | 0.8048 |
| rs748144 | 0.4490 | 0.4490 | 0.4950 | 0.3586 | 0.6414 | 0.3724 | 0.1466 | 0.4137 |
| rs7752055 | 0.0820 | 0.1630 | 0.1500 | 0.7268 | 0.2732 | 0.1387 | 0.0206 | 1.0000 |
| rs8072587 | 0.0560 | 0.1120 | 0.1060 | 0.8007 | 0.1993 | 0.1003 | 0.0104 | 1.0000 |
| rs830599 | 0.0770 | 0.1530 | 0.1410 | 0.7407 | 0.2593 | 0.1314 | 0.0183 | 1.0000 |
| rs885479 | 0.3880 | 0.5100 | 0.4750 | 0.4055 | 0.5945 | 0.3621 | 0.1966 | 0.5285 |

MAF, minor allelic frequency; Ho, observed heterozygosity; He, expected heterozygosity; MP, matching probability; PD, power of discrimination; PIC, polymorphism information content; PE, power of exclusion; *P_HWE_*, *P* values of 30 AISNPs for HWE tests in Xinjiang Hui group.
