# Supplementary figures and images for: Ancestry Prediction Comparisons of Different AISNPs for Five Continental Populations and Population Structure Dissection of the Xinjiang Hui Group via a Self-Developed Panel
Source: Genes (Basel). 2020 May 4;11(5):505. doi: 10.3390/genes11050505 (PMC7288656; doi:10.3390/genes11050505)

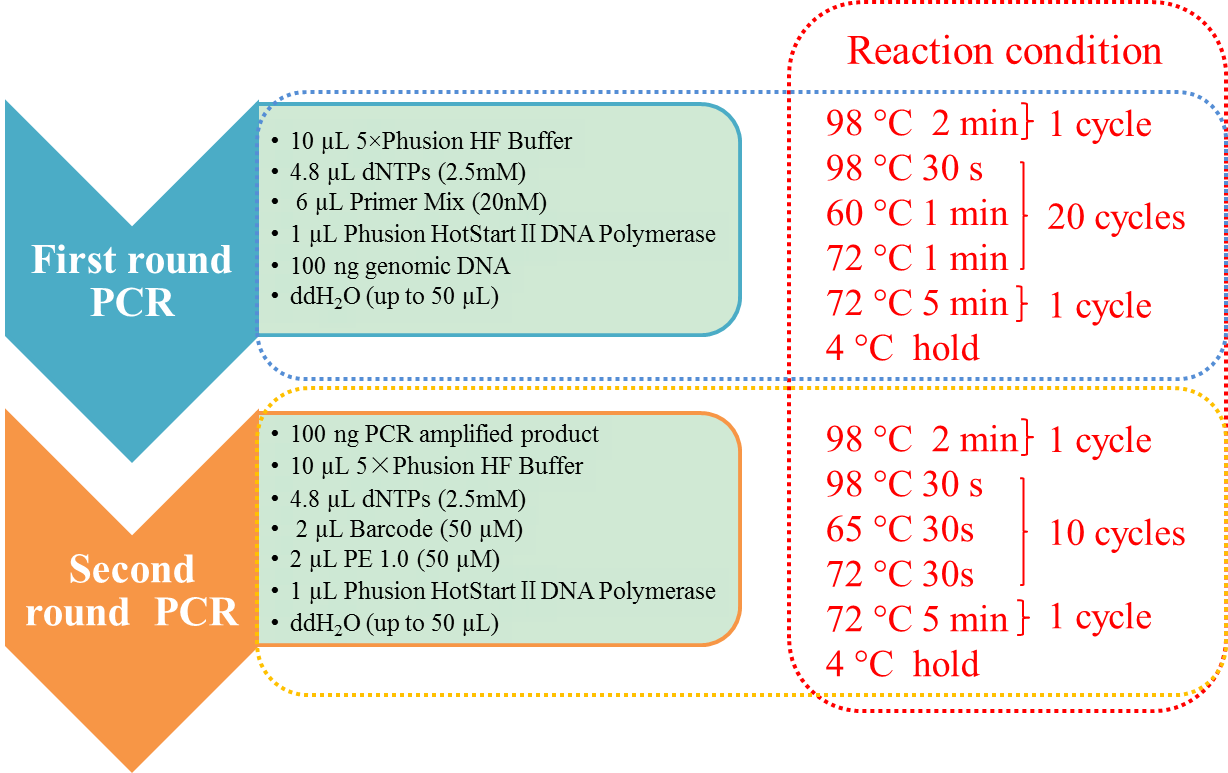

Supplement: Supplementary file 1 [file genes-11-00505-s001.zip › genes-734405-supplementary/Supplementary Figure S1.png]

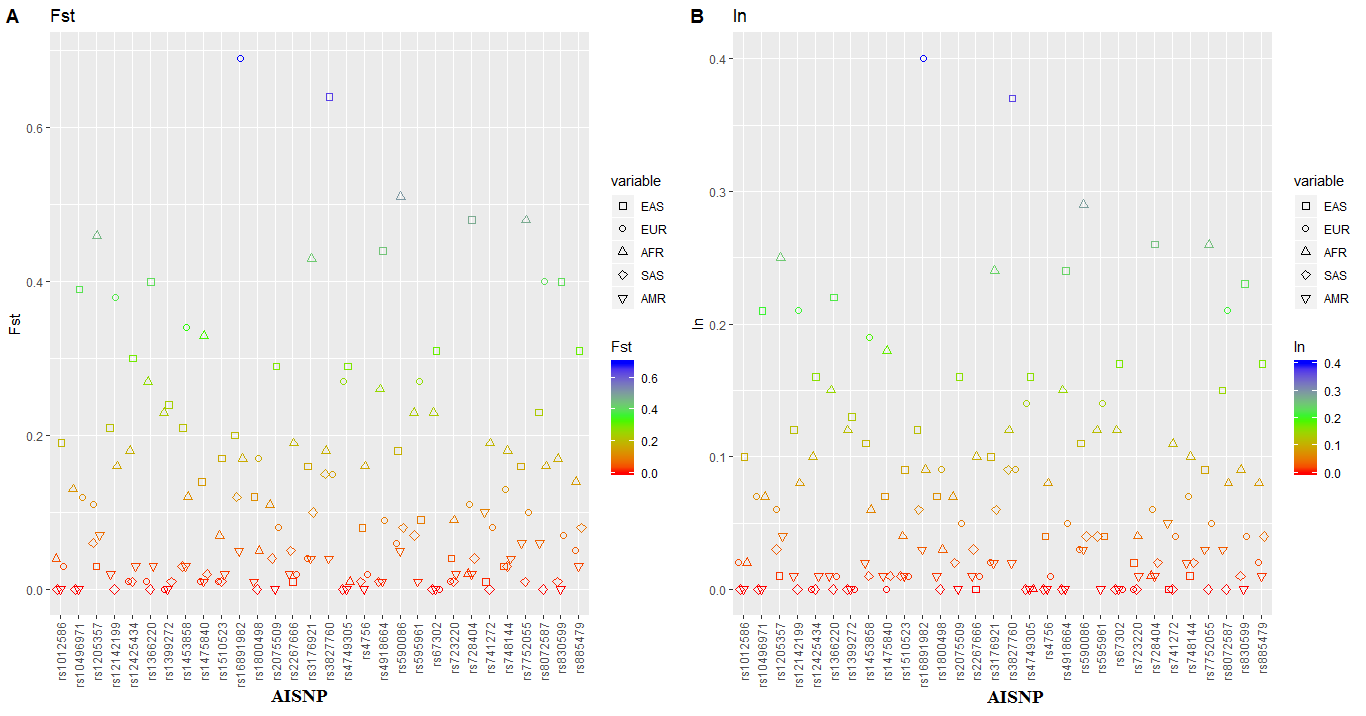

Supplement: Supplementary file 1 [file genes-11-00505-s001.zip › genes-734405-supplementary/Supplementary Figure S2.png]

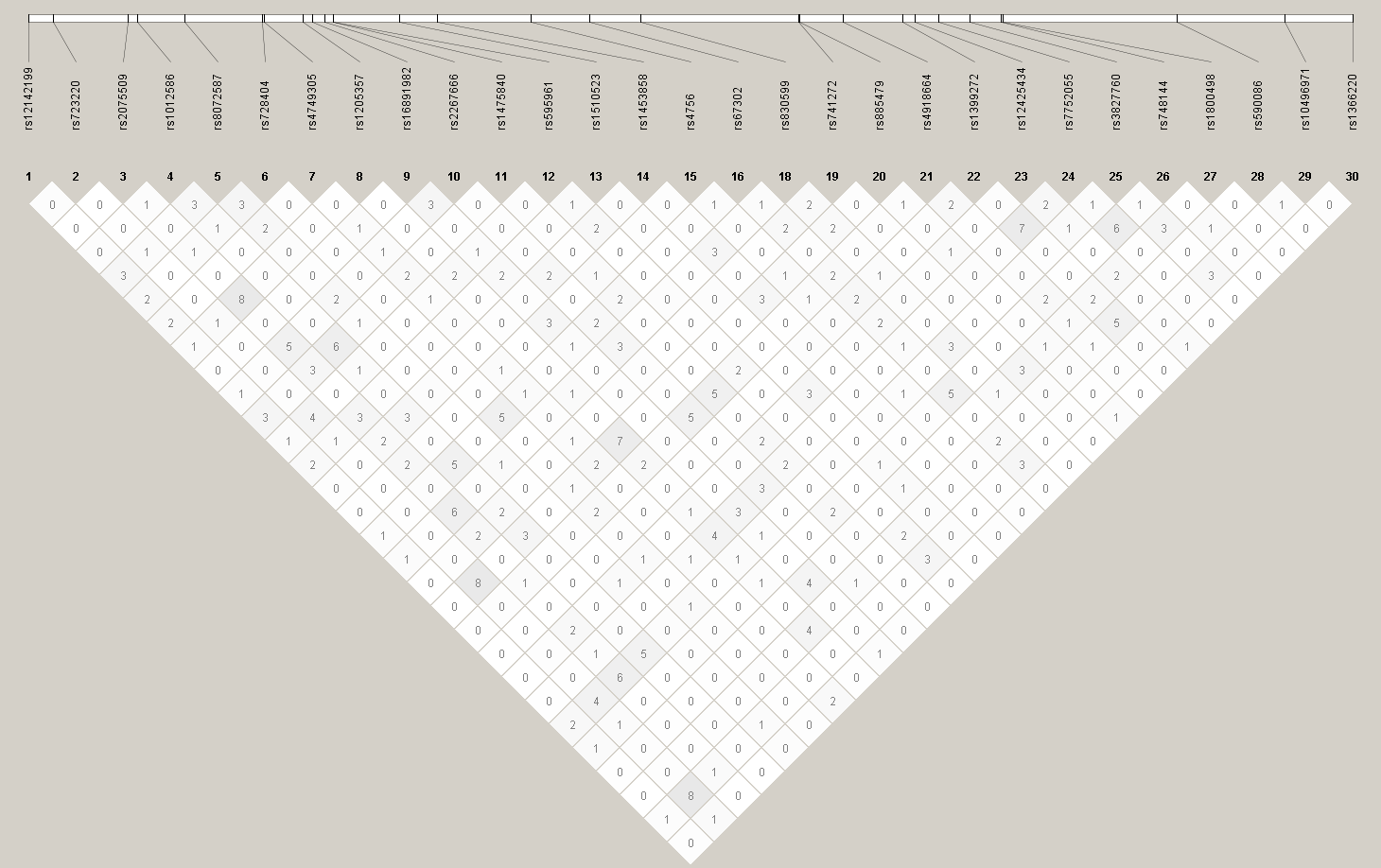

Supplement: Supplementary file 1 [file genes-11-00505-s001.zip › genes-734405-supplementary/Supplementary Figure S3.png]

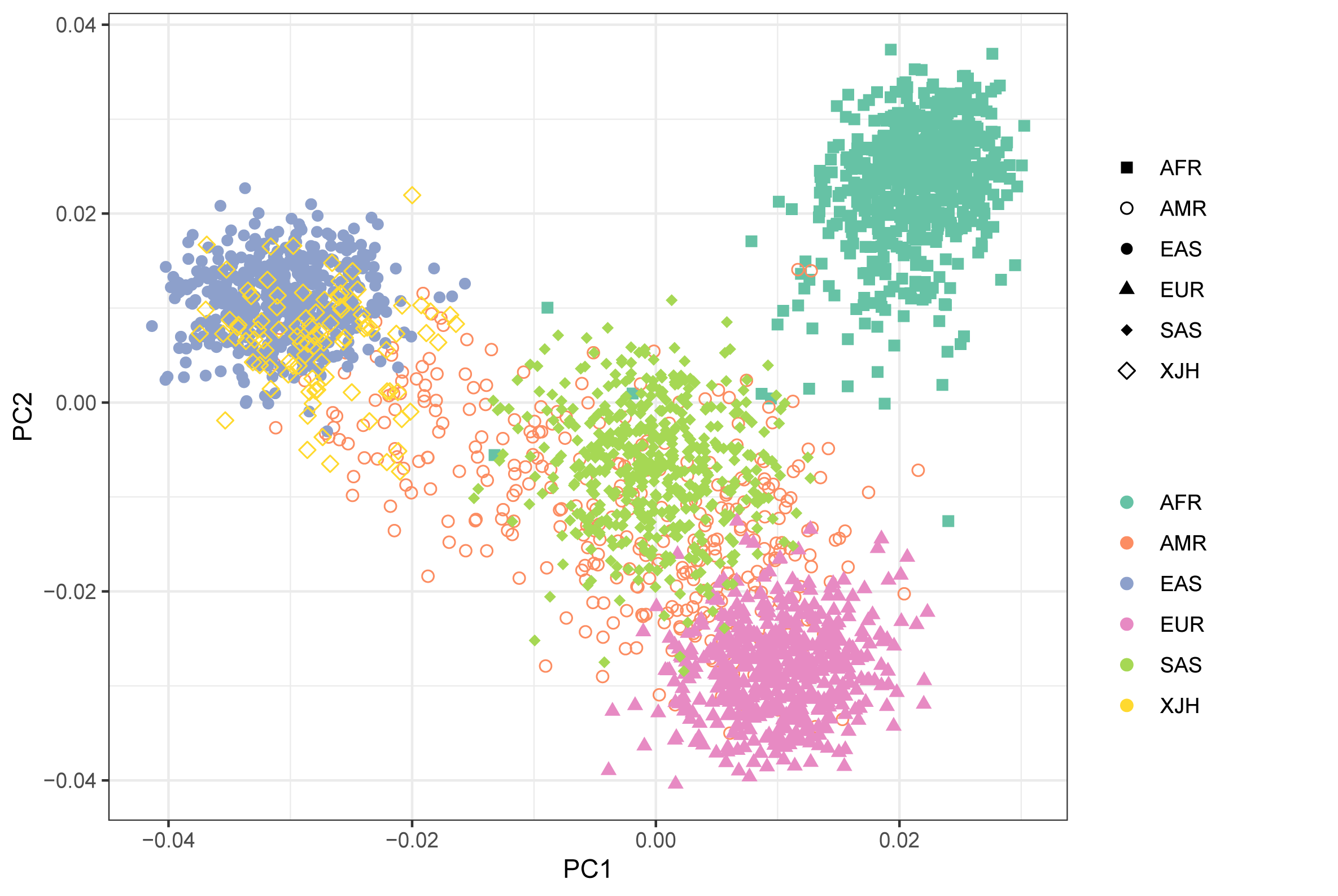

Supplement: Supplementary file 1 [file genes-11-00505-s001.zip › genes-734405-supplementary/Supplementary Figure S4.png]

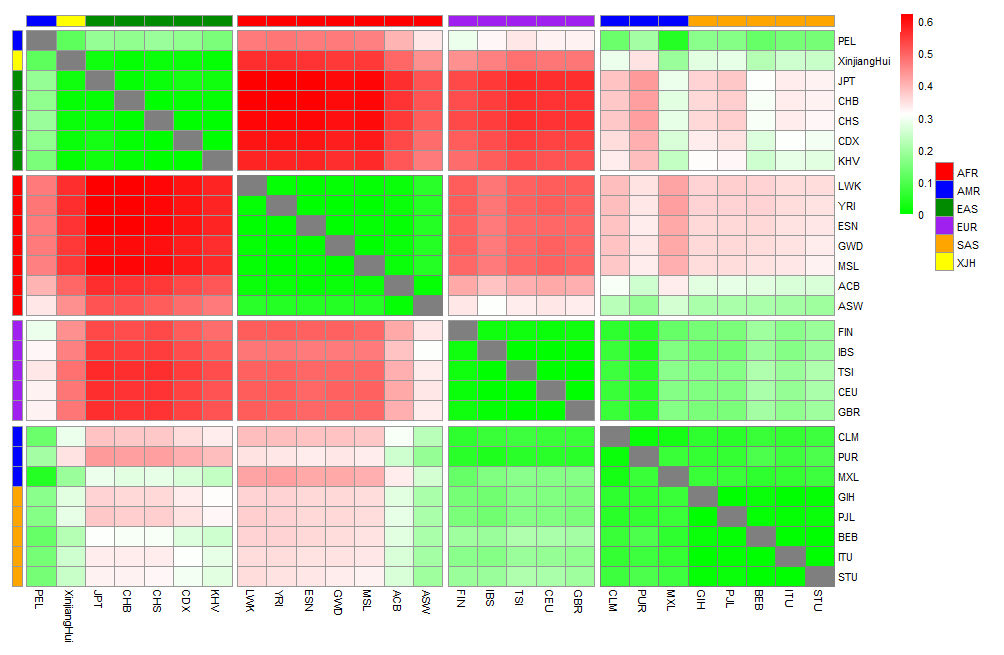

Supplement: Supplementary file 1 [file genes-11-00505-s001.zip › genes-734405-supplementary/Supplementary Figure S5.png]
